# Increasing allergy: are antibiotics the elephant in the room?

**DOI:** 10.1186/s13223-020-00432-2

**Published:** 2020-05-13

**Authors:** Rod A. Herman

**Affiliations:** Corteva Agriscience, 9330 Zionsville Road, Indianapolis, IN 46268 USA

**Keywords:** Antibiotics, Microbiome, Immune system, Immune disease, Allergy

## Abstract

Antibiotics cause dramatic changes to the human microbiome. The composition of the microbiome has been associated with changes in the immune system and these changes are beginning to be linked to immune diseases. Thus, antibiotics have been implicated as a significant contributor to the continual rise of allergies and autoimmune disease in developed countries. This recognition will hopefully result in the development of post-antibiotic therapies that restore a healthy microbiome and reduce immune system disorders.

## Antibiotics → microbiome → immune system → allergy

The problem of pathogen resistance in response to antibiotic use is well known as is the propensity of individuals to develop allergies to various classes of antibiotics [1–3]. Perhaps less widely known is that widespread antibiotic use in developed countries may be contributing to the observed general increase in the frequency of immune diseases, including food, respiratory, and dermal allergy [4–7]. Evidence linking the biodiversity and makeup of the microbiome to human health is accumulating including studies linking the microbiome (Box 1) to the proper functioning of our immune system [8–11]. Furthermore, a correlation between our microbiome characteristics and autoimmune disease and allergy has now been established [6, 12, 13]. It is therefore not surprising that a positive correlation between certain types of allergy and antibiotic use has been observed [4, 14, 15]. For example, the use of antibiotics in early childhood is associated with an increased rate of asthma and allergy (assessed at up to 12 years later) [16–18]. Although the tremendous benefits of antibiotics to fight pathogenic bacteria and improve the outcomes of disease is undeniable, antibiotics also kill many non-pathogenic bacteria. Furthermore, one would expect dramatic effects on the microbiomes in our lungs and gut, as well as on our skin occur during, and persist for a time after, a course of systemic antibiotics [19, 20]. Since our microbiomes play a role in allergy development, it should also not be surprising that antibiotic use can, through modification of our microbiomes, affect allergy development [4–6, 21]. While antibiotics are certainly not the only factors affecting our microbiome, understanding the intricacies of the effects of antibiotics on our microbiome and fostering the recovery of healthy microbiomes after antibiotic use remain challenges, and widespread appreciation of this phenomenon by researchers is likely key to fostering development of post-antibiotic therapies that do not perpetuate immune disorders [22]. With their widespread use and significant impact on the human microbiome, antibiotics may be the elephant in the room in terms of the increased frequency of allergy occurring in the developed world.

Box 1: Human microbiome The human microbiome refers to the microbial communities (bacteria, viruses, and fungi) that inhabit the human body including the gut, lungs, and skin (key sites of interaction between exogenous proteins and the immune system) [23]. The microbiome consists of the majority of the cells in the human body, and the sum of the genomes of these microbes is estimated at over 100-times greater than that of the human genome [4]. Humans and their microbiome have evolved together in often symbiotic relationships that facilitate good health [23, 24]. The makeup of the nascent microbiome is influenced by the mother (e.g., colonization from the vaginal microbiome) which can explain some immune diseases seen within families (e.g., asthma) [25]. However, whether specific changes to the microbiome cause disease, or are the result of disease, requires careful study. Environmental factors including diet are known to affect the composition of the microbiome, but perhaps no environmental factor has as dramatic an effect on the microbiome as antibiotics. Microbiome “transplants” from healthy to diseased individuals (e.g. fecal transplants) and probiotics are beginning to be used to treat certain diseases, including immune disease.

## Data Availability

Not applicable.
